# A reactor for time-resolved X-ray studies of nucleation and growth during solvothermal synthesis

**DOI:** 10.1107/S1600576723002339

**Published:** 2023-04-13

**Authors:** Martin Roelsgaard, Magnus Kløve, Rasmus Christensen, Andreas D. Bertelsen, Nils L. N. Broge, Innokenty Kantor, Daniel Risskov Sørensen, Ann-Christin Dippel, Soham Banerjee, Martin V. Zimmermann, Philipp Glaevecke, Olof Gutowski, Mads Ry Vogel Jørgensen, Bo Brummerstedt Iversen

**Affiliations:** a Department of Chemistry and iNANO, Aarhus University, Langelandsgade 140, 8000 Aarhus C, Denmark; b MAX IV Laboratory, Lund University, 224 84 Lund, Sweden; c Department of Physics, Technical University of Denmark, 2880 Kongens Lyngby, Denmark; d Deutsches Elektronen-Synchrotron DESY, Notkestraße 85, 22607 Hamburg, Germany; Instituto Andaluz de Ciencias de la Tierra, Granada, Spain

**Keywords:** *in situ* X-ray scattering, powder X-ray diffraction, total scattering, small-angle scattering, solvothermal chemistry

## Abstract

Understanding the nucleation and growth mechanisms of nanocrystals under hydro- and solvothermal conditions is key to tailoring functional nanomaterials. High-energy and high-flux synchrotron radiation is ideal for characterization by powder X-ray diffraction and X-ray total scattering in real time. Different versions of batch-type cell reactors have been employed in this work, exploiting the robustness of polyimide-coated fused quartz tubes with an inner diameter of 0.7 mm, as they can withstand pressures up to 250 bar and temperatures up to 723 K for several hours. Reported here are recent developments of the *in situ* setups available for general users on the P21.1 beamline at PETRA III and the DanMAX beamline at MAX IV to study nucleation and growth phenomena in solvothermal synthesis. It is shown that data suitable for both reciprocal-space Rietveld refinement and direct-space pair distribution function refinement can be obtained on a timescale of 4 ms.

## Abstract 2

A versatile reactor for *in situ* X-ray scattering studies of solvothermal reactions is presented, capable of providing data with millisecond time resolution

## 1. Introduction

Solvothermal synthesis is a versatile technique that is widely used to synthesize nano-sized inorganic materials. The term ‘hydro­thermal’ covers those reactions where the solvent is water. As the name implies, it is defined as a process in a closed reaction vessel inducing a decomposition or a chemical reaction between precursor(s) in the presence of a solvent under elevated pressure and temperature to obtain a reaction product (Demazeau, 2010 ▸). The temperature and pressure span a wide range including both sub- and supercritical water (*P*_c_ = 221 bar, *T*_c_ = 647 K; 1 bar = 100 000 Pa). Hydro- and solvothermal synthesis are fast and cost-effective techniques which are also scalable, *e.g.* in versatile flow reactors (Hellstern *et al.*, 2016 ▸; Aymonier *et al.*, 2018 ▸; Hald *et al.*, 2006 ▸). Investigating and gaining understanding of the reaction mechanisms under these conditions becomes a challenge, in part due to the boundaries of the reaction vessel being a black box in the laboratory setting and in part due to the often rapid nature of the chemical reactions. Conventional laboratory methods are mostly limited to *post mortem* and *ex situ* studies, *i.e.* either limited to studying the final products or quenching the reaction at various reaction times.

Microstructural information can be obtained through *in situ* X-ray experiments, utilizing the high-flux and high-energy X-ray beams found at synchrotron radiation facilities which can penetrate specialized reaction vessels while retaining a temporal resolution on the relevant time scales. Some of the first applications of synchrotron X-ray diffraction to study hydro­thermal reactions investigated the crystallization of zeolites (Norby, 1997 ▸; Norby *et al.*, 1994 ▸), and Jensen *et al.* (2014 ▸) have provided a review of *in situ* studies. Simultaneous *in situ* powder X-ray diffraction (PXRD) and small-angle X-ray scattering (SAXS) studies of titania nanoparticle formation under supercritical conditions were pioneered by Jensen *et al.* (2007 ▸) at the Advanced Photon Source (Argonne National Laboratory, Illinois, USA), followed by a study of magnetite (Bremholm, Felicissimo & Iversen, 2009 ▸). Amorphous or disordered components during solvothermal synthesis can be studied with *in situ* X-ray total scattering (TS) and pair distribution function (PDF) analysis, as shown by Bremholm, Becker-Christensen & Iversen (2009 ▸) in studies of zirconia nanoparticles at the MAX II facility (Lund University, Sweden).

Becker *et al.* (2010 ▸) developed the first version of the experimental reactor setup used in the present work for *in situ* SAXS, PXRD and PDF studies of hydro- and solvothermal reactions. Other reactors exist, as reviewed by Jensen *et al.* (2014 ▸), and the various reactors have different strengths including probing at the mixing point of a mixing-flow reactor (Beauvais *et al.*, 2022 ▸). Alternative approaches include employing nuclear magnetic resonance tubes with a larger diameter of 3 mm, such as in a recent study of PdCu_3_ formation (Mathiesen *et al.*, 2022 ▸).

The reactor forming the basis of this work has been employed in numerous studies and only a few examples will be mentioned. The first quantitative modelling of *in situ* PDFs obtained from solvothermal reactions was a study of SnO_2_ formation from SnCl_4_ (Jensen *et al.*, 2012 ▸) and CeO_2_ from Ce(NH_4_)_2_(NO_3_)_6_ precursor (Tyrsted *et al.*, 2012 ▸). Bøjesen & Iversen (2016 ▸) have reviewed such *in situ* PDF studies. The many adjustments to the original reactor that have led to the present report were in large part carried out at PETRA III (DESY, Hamburg, Germany), which provides a high-energy X-ray beam for easy reactor penetration and fast data acquisition (Lindahl Christiansen *et al.*, 2020 ▸; Billinge, 2019 ▸; Terban & Billinge, 2022 ▸). Important studies at PETRA III leading to the present reactor design include the formation mechanisms of WO_3_ (Saha *et al.*, 2014 ▸) and ZnWO_4_ (Bøjesen *et al.*, 2016 ▸), polymorphism in ZrO_2_ (Dippel *et al.*, 2016 ▸) and HfO_2_ (Christensen *et al.*, 2021 ▸), proof of the secondary building unit hypotheses of metal–organic frameworks (Xu *et al.*, 2019 ▸), structural characterization of metal ion complexes in solution (Nielsen *et al.*, 2021 ▸; Kløve *et al.*, 2022 ▸), establishing the redox chemistry of solvents under high-pressure and high-temperature conditions (Broge, Søndergaard-Pedersen *et al.*, 2020 ▸), and establishing the autocatalytic formation of high-entropy alloys from acetyl acetonates of Ru, Rh, Ir, Pd and Pt (Broge, Bondesgaard *et al.*, 2020 ▸). Andersen *et al.* (2018 ▸) discussed the pitfalls and reproducibility of this type of study, in particular for powder diffraction studies.

A wide range of changes have been made to the setup described by Becker *et al.* (2010 ▸), which this work attempts to collect and report. This includes a reactor with minor modifications to employ an in-house Rigaku SmartLab X-ray source (Gjørup *et al.*, 2019 ▸; Sun *et al.*, 2019 ▸), with the benefit of completely avoiding the access and time constraints on synchrotron beamlines. For synchrotron X-ray sources, the reactor has been implemented on the side station of the Swedish Materials Science beamline P21.1 at PETRA III, and on DanMAX at MAX IV. The P21.1 endstation is a canted beamline operating at 52, 85 and 103 keV photon energies with a flux of 2 × 10^11^ photons s^−1^ at 103 keV with the single-bounce monochromator. DanMAX is tunable in the energy range 15–35 keV with a flux between 4 × 10^13^ photons s^−1^ (15 keV) and 5 × 10^12^ photons s^−1^ (35 keV) with a horizontal double-crystal monochromator. P21.1 has an option to choose between a PerkinElmer XRD1621 area detector and a DECTRIS PILATUS3 X 2M CdTe detector from a shared pool, while DanMAX exclusively employs a PILATUS3 X 2M CdTe detector.

With the high photon energies of the P21.1 beamline and an area detector, TS data quality for PDF analysis is readily obtained with second-scale temporal resolution. DanMAX operates at lower photon energies, requiring smaller sample-to-detector distances to obtain PDFs, which is challenging with bulky equipment. Generally, a finite *Q*_max_ value results in spurious oscillations in the PDF which are usually further enhanced by a poor signal-to-noise ratio at high *Q* values [*Q* = (4π/λ)sin(θ/2), where θ is the scattering angle and λ is the wavelength of the incident radiation]. The smallest meaningful value in Δ*r* is given by the Nyquist–Shannon theorem, *i.e.* Δ*r* = π/*Q*_max_ (Terban & Billinge, 2022 ▸; Farrow *et al.*, 2011 ▸), as is demonstrated in Fig. S5 in the supporting information. A lower achievable *Q*_max_ results in broader PDF peaks that may merge. Such analysis is exemplified by in-depth studies of precursor structures in solution prior to and in the early stages of solvothermal synthesis (Kløve *et al.*, 2022 ▸; Sommer *et al.*, 2020 ▸; Nielsen *et al.*, 2021 ▸). On the other hand, DanMAX excels with very high intensity at lower photon energies for *in situ* PXRD studies while still yielding good PDF quality when information on the short-range order is required. This makes it possible to gain insight into structural transformations and reaction mechanisms on a sub-second timescale which is explicitly highlighted in this work. Overall, the two beamlines offer high resolution either in time or in real space and thus ideally complement each other for *in situ* solvothermal studies.

## 2. Reactor cell design

### 2.1. Reactor cell materials

**Figure 1:**
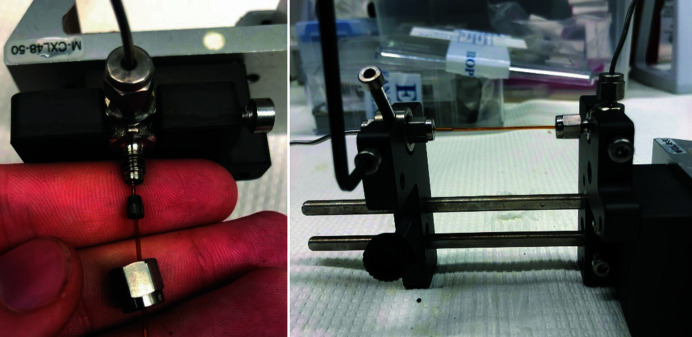
Photographs depicting (left) the assembly of 0.85 mm (Ø OD) fused quartz with polyimide coating, graphite ferrule for 1/16′′ with 1.0 mm hole and 1/16′′ Swagelok elbow fitting on the optical rail carrier side, and (right) fastening of the Swagelok fixture in the completed reactor cell.

The reactor employs 0.7 mm inner diameter (0.85 mm outer diameter, OD) fused quartz tubes with an outer polyimide coating, which are readily commercially available for gas chromatography systems as long columns. The tubes are cut to approximately 7–8 cm in length and fastened in Swagelok 1/16′′ fittings (typically with an elbow inboard to the storage ring and a straight at the outboard side) by either graphite or Teflon ferrules. The assembly is depicted in Fig. 1 ▸ with a graphite ferrule and a 1.0 mm bore that is fastened to form a tight seal up to pressures of 250–260 bar. Each completed cell is pressure tested with water to avoid chemical spillage at the experimental end station. Towards the optical rail carrier side the Swagelok elbow piece is directly fixed in place by a fastening screw, whereas the straight 1/16–1/16′′ piece is fixed in place using a stainless steel cylinder with a threaded hole. This gives additional flexibility for releasing strain through rotation while deforming the ferrule, thus minimizing the risk of rupture of the capillary when external pressure is applied.

The fused quartz tubes withstand a pressure of 250 bar and temperatures up to approximately 723 K, sufficient to reach supercritical conditions of water (*P*_c_ = 221 bar, *T*_c_ = 647 K). Hollow sapphire tubes sustain pressures surpassing 300 bar (commercially available for example from Crytur), with inner diameter and outer diameter ranging from 0.50 to 0.75 mm, and 1.2 mm to 2.0 mm, respectively. However, the strong single-crystal scattering from these tubes is challenging for the dynamic range of large-area detectors and difficult to reproduce between experiments, making the scattering in those regions sacrificial. The main benefit from a chemical point of view is that sapphire can withstand strong alkaline conditions. However, in the case of TS experiments that typically involve Fourier analysis of the azimuthally integrated scattering pattern, thermal diffuse scattering from the sapphire tube is disadvantageous. It is not sufficient to mask away the regions around the Bragg reflections of the sapphire tube. The fused quartz tubes are amorphous and thus better suited for TS, as they give rise to a scattering pattern that is homogeneous around the azimuth. This scattering pattern is virtually impossible to mask but readily subtracted, either in raw 2D format or, more commonly, in 1D format following azimuthal integration, provided that a second reference experiment is collected at each of the desired synthesis temperatures. Note that the reactivity of the tube material towards the chemicals in the precursor should be taken into account.

The precursor is injected via a standard syringe into 1/16′′ Swagelok fittings. At the beamline endstation the cell is mounted onto optical rails from Newport (X48 optical rails) that slide along the vertical axis, to be as modular as possible. For example, using this system it is possible to perform ‘mixing experiments’ with syringe pumps and lines entering through a Swagelok T-piece, albeit not under pressure (Birgisson, 2017 ▸). For mounting a cell, a secondary empty rail carrier is used as a stop block when inserting the sample, ensuring a reproducible sample position and avoiding collision with the nozzle of the heater.

**Figure 2:**
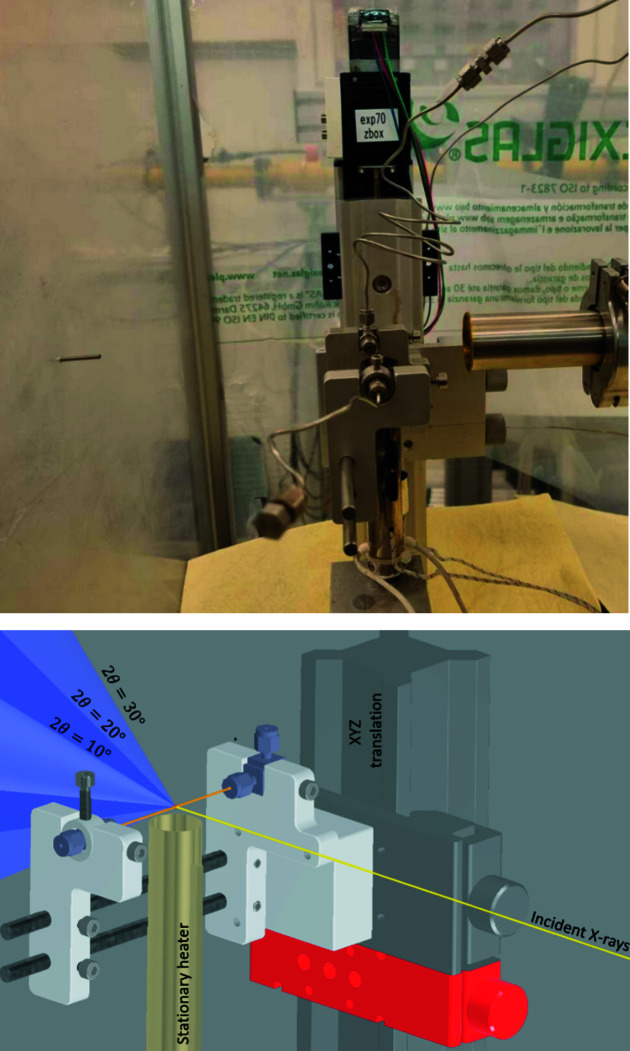
(Top) Photograph of the cell installed on the P21.1 beamline. (Bottom) A CAD rendering with the heater assembly centred 6 mm below the sample. The incident X-ray beam is depicted as a yellow line, with Debye–Scherrer cones from the capillary at 10, 20 and 30° in 2θ.

The heater and reactor cells have varying adapters between the beamlines but are otherwise compatible with each other. Fig. 2 ▸ shows a side-view depiction of the cell installed on P21.1, as well as a computer-aided design (CAD) depiction showing the incident X-ray beam and diffraction cones at 10, 20 and 30° (in 2θ).

### 2.2. Heating and pressurization

The reactor developed by Becker *et al.* (2010 ▸) deployed a commercial air heater (Leister, LHS-20L) with a branched outlet operated by a pneumatic shutter system to pre-heat the air with proportional integral derivative (PID) control prior to commencing an experiment. The heated air shutter system helped to ensure a fast transition to the target temperature. The temperature was maintained by a thermocouple situated just in front of the branching and controlled by a commercial PID controller, albeit lacking integration into the beamline controls, which was implemented as the reactor was adapted for the current setup on P21.1.

In order to simplify and miniaturize the setup, the heater was exchanged for a constant air stream with direct heating, *i.e.* without the pneumatic shutter system (Becker *et al.*, 2010 ▸). Heat is applied in a reaction zone approximately 13 mm wide by a 60 V direct current (DC) heating system commercially available from Hybec A/S (HiHeater type A 500 W), with a thermocouple in line with the heater outlet. The heater nozzle is placed 6 mm below the sample to avoid the steel housing shadowing the scattered X-ray signal downstream towards the detector. An SMC gas flow controller is used to control the air supply and delivers a constant flow in the range 10–100 L min^−1^ of pressurized air to the heater. This is also coupled to the power supply of the heater, to ensure power shut-off as a safety measure if the airflow is interrupted. The secondary thermocouple at the bottom of the steel housing is monitored and automatically interrupts the power supply if a temperature of 423 K is reached, to avoid overheating of the heater.

The power is delivered by an ElektroAutomatik PSI 5080 20 A power supply that is controlled by a connection to the analogue output of a Lakeshore 336 PID controller, which is connected to the thermocouple at the outlet of the heater. The PID controller supports both immediate ‘jumps’ in temperature set points to emulate high heating rates, *e.g.* similar to the injection of precursors into a hot liquid, and gradual ramping over time with a rate up to 100 K min^−1^ to the target temperature, or more complicated sequenced temperature programs. Furthermore, on the beamlines at PETRA III and MAX IV the heater setup has been integrated into beamline controls via the *Tango Controls* environments (https://www.tango-controls.org/) used at the facilities. Temperature values are thus readily saved in the measurement metadata during the experiment.

A temperature calibration is usually performed at the beginning of each beam time to determine the offset in temperature measured by the thermocouple in the airstream (which is used by the PID controller) and the temperature inside the capillary. A thermocouple (steel-sheathed type K) with diameter of 0.25 or 0.50 mm is inserted into the capillary using a T-piece Swagelok connector and a smaller bore graphite ferrule on the thermocouple side. During the calibration the reactor can be empty (*i.e.* filled with air) or under hydro­static pressure, as the response is not significantly altered (see Fig. S1). Note that fused silica tubes are prone to rupture with an internal thermocouple. Another approach is to perform the calibration exploiting the thermal expansion of a well known crystalline material with X-rays, such as hexagonal BN (Marshall *et al.*, 2020 ▸).

**Figure 3:**
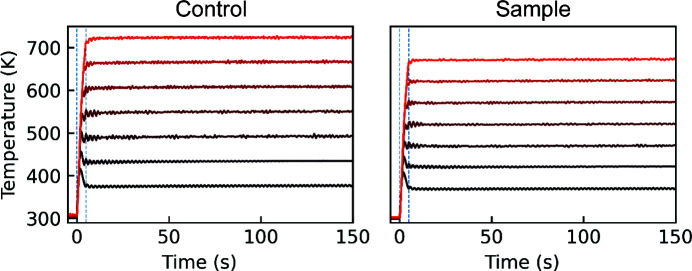
Heater response curves as measured (left) by the heater and (right) by a thermocouple inside the tube, in 50 K increments from 373 to 673 K at the sample. The PID settings were chosen to reach a flat plateau quickly at the desired reaction temperature inside the tube, taking the offset between the measured air temperature at the heater nozzle (left) and at the beam position inside the tube (right) into account. The dashed vertical lines are placed at *t* = 0 and 5 s.

The temperature offset is dependent on the airflow, *i.e.* with a relatively high airflow (*e.g.* 40 L min^−1^) the offset approaches 10% of the applied heat at 673 K target temperature. Therefore, to reach that temperature a setpoint of 713 K is set on the controller. With PID settings calibrated (which are highly dependent on the heater–reactor distance and the airflow) for 20 L min^−1^ with a minor overshoot at high temperature, the temperature curves seen in Fig. 3 ▸ illustrate this for the direct (fastest) step to a setpoint temperature from room temperature. At the lower end of the flow rates the heater is also capable of achieving temperatures of 973 K, although this has not seen any application within the scope of solvothermal reactions.

Hydro­static pressure is applied by a LabAlliance PrepPump through a steel tube. The pump is controlled either in person when preparing an experiment or remotely after setting the interlock, through the RS232 serial connection that has been implemented in *Tango Controls* for monitoring during the experiment. The temperature, pressure, monitor reading for the X-ray flux and other experimental information is also polled for ease of troubleshooting at a later stage, usually at a rate of 1–4 Hz.

### 2.3. Safety aspects

The main safety aspect of running such a reaction is the risk of rupture, which is a relatively rare event even for supercritical reactions. The cell is thus placed inside a splash box to inhibit direct line of sight from the outside, such that any splashes are caught and can be cleaned up accordingly. Most faults occur during assembly, *i.e.* prior to working with precursors. However, some precursors can degrade the reactor walls, such as highly alkaline solutions or materials with a high reactivity towards the reactor wall like manganese-forming manganese silicates, and these conditions may result in rupture of the capillaries.

The reactor cells are typically operated in the range 30–250 bar and heated locally near the exhaust of the heater, to ensure a subcritical liquid or a supercritical phase within the reactor. Without the externally applied pressure most solvents would evaporate and form internal gas cavities (bubbles), since the entire volume of the pressurized system is not heated. Phase changes and high diffusion causing the addition of new material into the probed region are also undesirable for the reaction.

On the P21.1 endstation at PETRA III the enclosure is mounted on a general-purpose diffractometer on the beamline. An evacuated flight tube protrudes into the splash box from the upstream side, and this is used to collimate the beam and minimize scattering from air before the X-ray beam reaches the sample. The beamstop is installed directly into a threaded hole in the solid poly(methyl methacrylate) (PMMA) sheet on the exit window side of the splash box, thus requiring translation of the whole box in order to centre the beamstop on the incident X-ray beam. Additionally, the reactor is centred above the heater and in the incident X-ray beam using an *xyz* stage mounted inside the splash box. An optical camera is mounted above the cell looking down into the heater (protected from the heated air behind PMMA or glass). The camera aids the alignment and readily allows reproducible positioning of the reactor at a precision of 0.1 mm in the up-/downstream direction and hence assures an identical sample-to-detector distance after sample change. To centre the reactor in the vertical direction it is translated while monitoring the scattering intensity on the area detector (which is preferred over inserting a diode setup inside the splash box in front of the beamstop for practical reasons, as well as for a better signal from scattering than from absorption at high photon energies).

On DanMAX both the beamstop and beam pipe protrude into the enclosure. Furthermore, the sample is mounted directly onto the box (on a Newport X48 optical rail carrier), thus requiring relatively large holes in the PMMA shielding to encompass translations. The splash box and reactor cell are mounted on a heavy-duty hexapod that facilitates alignment of the sample to the X-ray beam. Towards the detector side a cutout is covered in two layers of polyimide (175 µm thickness each), as a large fraction of the lower photon energies would be absorbed in the thicker PMMA. The polyimide is situated approximately 70 mm downstream of the sample. Chemical absorbent pads are cut and installed over the openings (at the heater, beamstop and beam pipe) to avoid direct line of sight to the reactor from the outside.

During sample preparation a volume of less than 0.5 ml of precursor solution is injected into the cell, which is sealed in a chemistry laboratory prior to being transported to the endstation. For reactions that are sensitive to atmospheric exposure, it is possible to prepare the cells in a glove box.

## 3. Time-resolved PDF studies at 35 keV

Time-resolved PDF studies with time resolution on the millisecond scale were demonstrated using the *in situ* reactor deployed on the DanMAX beamline at MAX IV, with the synthesis of HfO_2_ nanoparticles as a case study. This synthesis was previously investigated in further detail (Christensen *et al.*, 2021 ▸) on the P21.1 beamline (PETRA III) with an energy of 103 keV and a PerkinElmer XRD1621 a-Si detector. Notably, from this study the nucleation and growth event of the HfO_2_ nanoparticles seemed almost instantaneous at temperatures above 673 K, since only a single data point on the steepest part of the growth curves was available. Here, we briefly revisit the synthesis to demonstrate the feasibility of performing PDF studies with millisecond-scale time resolution to unravel nucleation and growth events that happened within a single frame in the previous study. The highly efficient hybrid photon-counting detection with low noise provided by the DECTRIS PILATUS3 X 2M CdTe detector, high incident photon flux and lower photon energy (and thus higher scattering power) are all beneficial factors in improving the temporal resolution in the relevant time interval that is possible in the experiment.

**Figure 4:**
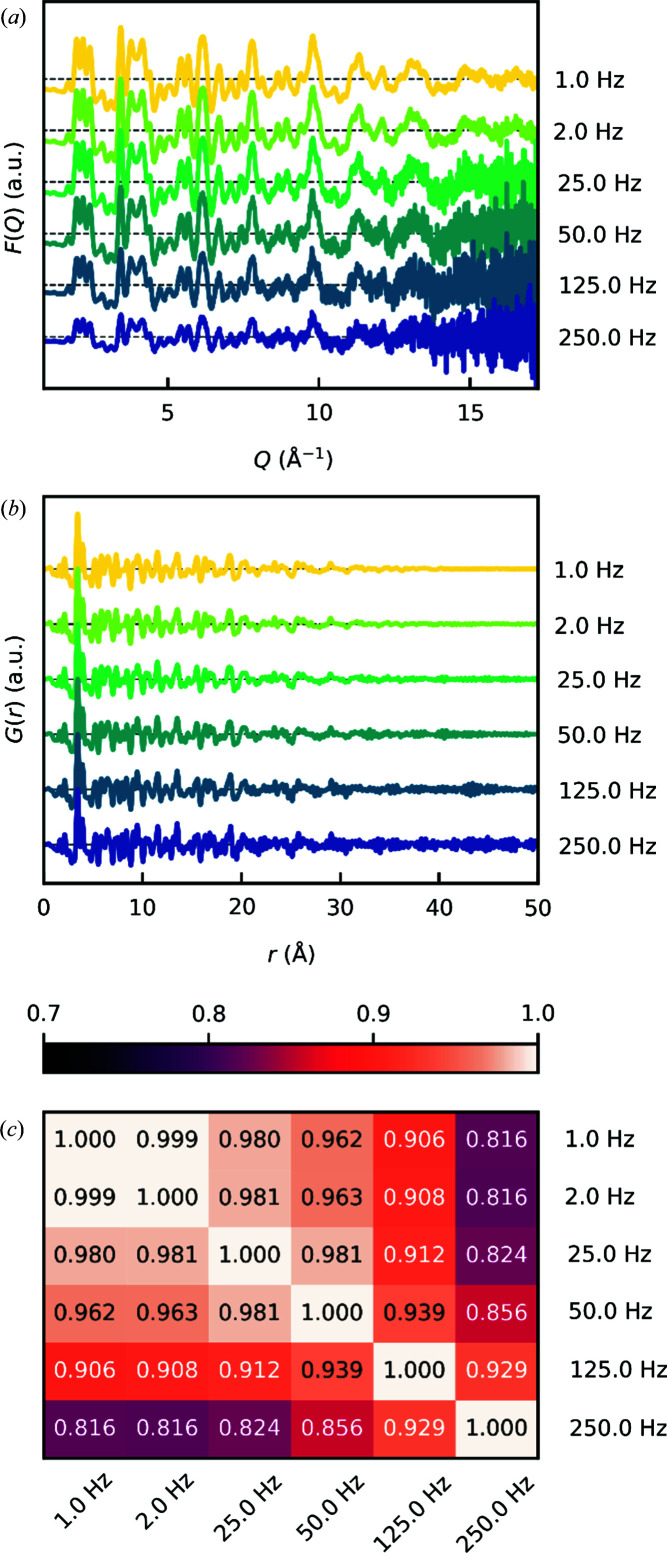
Data from an *in situ* experiment performed on DanMAX on the nucleation and growth of HfO_2_ nanoparticles obtained at a 250 Hz acquisition rate and summed to simulate data collection rates in the range 1–250 Hz. (*a*) Reduced structure functions *F*(*Q*). (*b*) PDFs *G*(*r*). (*c*) A Pearson correlation coefficient matrix comparing the linear correlation between the PDFs from panel (*b*) in the range 0–50 Å.

A synthesis equivalent to that of Christensen *et al.*, *i.e.* a 1 mol L^−1^ solution of HfCl_4_ in methanol, pressurized to 250 bar and subsequently heated to the desired temperature of 723 K, was carried out while collecting scattering data. The acquisition time was 0.004 s (X-ray exposure of 0.003 s and readout time of 0.001 s), *i.e.* a collection frequency of 250 Hz (Fig. 4 ▸). While the data were collected with a total acquisition time of 0.004 s per frame, we summed these frames to simulate data collected in the range 1–250 Hz for the analysis below. It is noted that Hf has a high scattering power owing to its high atomic number, which is beneficial towards short acquisition times. For systems of lower scattering power and lower precursor concentration, the time resolution may be correspondingly lower.

In Figs. 4 ▸(*a*) and 4 ▸(*b*), the quality of the reduced structure function *F*(*Q*) and the PDF *G*(*r*), generated with a *Q*_max_ of 17.2 Å^−1^, is shown. Evidently, the noise level in the high-*Q* region of *F*(*Q*) becomes quite significant at very short exposures.

With the present detector configuration, an instrumental *Q*_max_ of 17.2 Å^−1^ can be achieved. A *Q*_max_ in the region of 17–25 Å^−1^ is common for these types of experiments that involve non-ideal experimental conditions, such as rapid acquisition but also, in particular, dynamic solution structures at elevated temperatures. However, even lower values of *Q*_max_ can be tolerated depending on the scientific questions one seeks to answer.

As a means of evaluating the quality of the PDF, a Pearson correlation coefficient matrix was calculated for the PDFs [Fig. 4 ▸(*c*)]. The Pearson correlation coefficient is a measure of the linear correlation between two data sets. With coefficients above 0.8, all PDFs are clearly correlated and must therefore basically convey the same informational content. Evidently, the coefficients gradually decrease with increasing noise in the PDF, showing a strong bias for the coefficients to noise.

**Figure 5:**
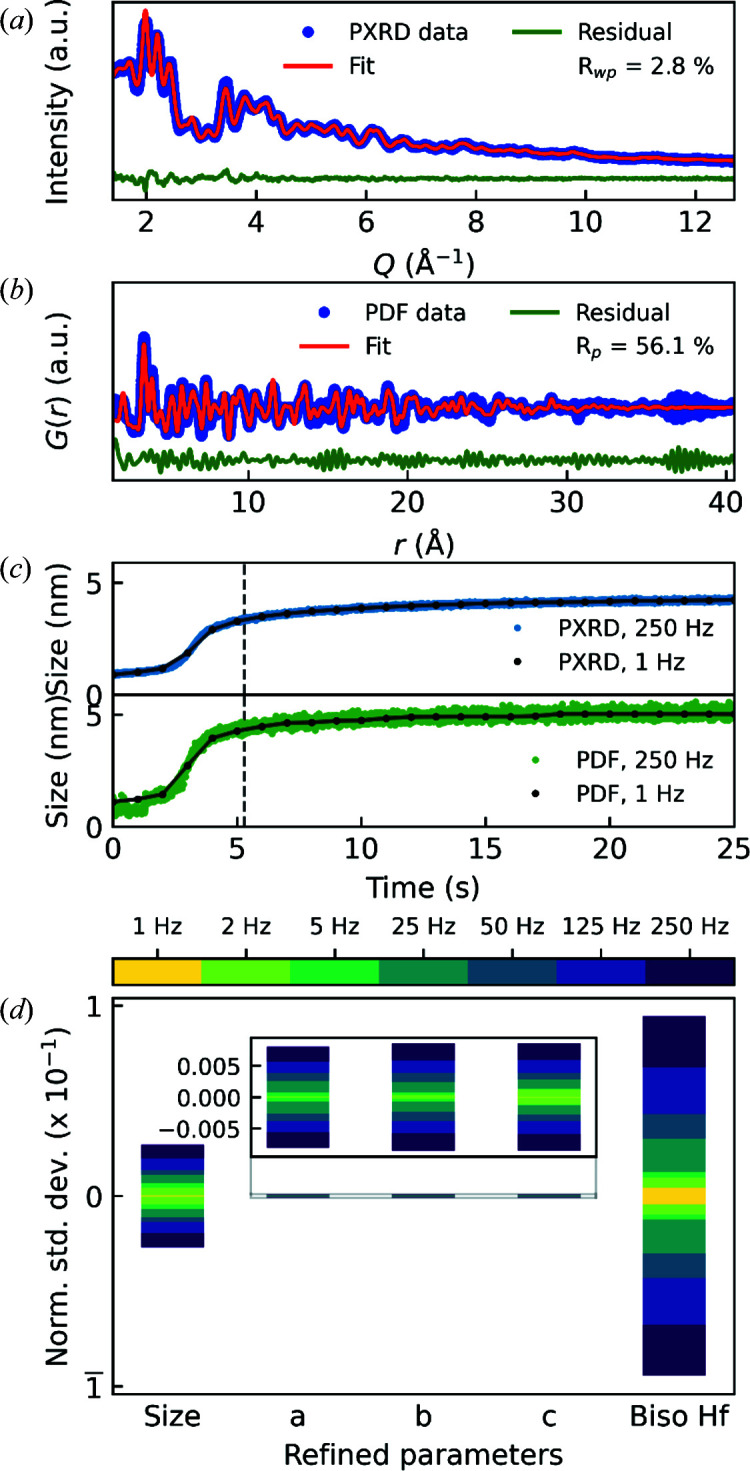
Structural refinements on (*a*) 250 Hz reciprocal-space (PXRD) data (*R*_wp_ = 2.8%) and (*b*) 250 Hz real-space (PDF) data (*R*_p_ = 56.1%) after ∼5.3 s. (*c*) Average crystallite sizes obtained from Rietveld refinements on 250 Hz PXRD data (top, blue dots) and average coherently scattering domain sizes obtained from real-space refinements on 250 Hz PDF data (bottom, green dots). Refinements were performed on 1 Hz reciprocal-space and PDF data as well. Refined average crystallite sizes and average coherently scattering domain sizes from these refinements are overlaid on the respective curves with black dots. (*d*) Normalized standard deviations on refined parameters from refinements on 1–250 Hz PDFs in the time interval 20–25 s. The refined values are normalized to the mean within the time interval by *x*_norm_ = 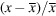 and the normalized standard deviation is subsequently calculated as σ_norm_ = 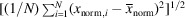 .

The data obtained at 250 Hz were subject to both Rietveld refinement in reciprocal space (PXRD) and PDF refinement in real space (Fig. 5 ▸). Examples of the refinement results are shown in Figs. 5 ▸(*a*) and 5 ▸(*b*), respectively. Both refinements confirm that the sample indeed consists of monoclinic HfO_2_ nanocrystals. The reciprocal-space data are particularly well described by the model and they result in a low agreement factor *R*_wp_ of 2.8%. It is a great testament to the high-intensity beam of DanMAX and the low detection noise that a diffraction pattern collected at 250 Hz with applied heat on a suspension of quite small nanoparticles can be modelled with such agreement. The refinement of the 250 Hz PDF, on the other hand, is heavily affected by the increasing noise level in the high-*Q* region of *F*(*Q*), which is discarded in the reciprocal-space refinements due to the lack of visible Bragg peaks, and results in a quite high agreement factor *R*_p_ of 56.1%. Still, all the structural features of the PDF can be modelled, which results in a difference curve mostly consisting of high-frequency noise. Notably, the calculated PDFs resulting from fitting the 250 and 1 Hz PDFs are almost identical, though the *R*_p_ is greatly reduced when noise is minimized (Fig. S2).

The refined particle sizes (average crystallite sizes for PXRD and average coherently scattering domain sizes for PDF) show a similar temporal trend regardless of refinement method (PXRD versus PDF), although absolute values differ between the methods. This is expected when comparing PXRD and PDF data and was also observed in the original study (Christensen *et al.*, 2021 ▸). Notably, the spread of the PDF sizes is significantly larger than those obtained from PXRD. Taking the mean and standard deviation of the refined sizes in the time interval of 24–25 s gives 4.22 (4) and 5.07 (13) nm for PXRD and PDF data, respectively, *i.e.* a standard deviation more than three times larger for the PDF. By summing consecutive frames, the spread of refined values is reduced since noise and temporal changes are averaged out [1 Hz curves in Fig. 5 ▸(*c*) and Fig. S3].

The variance of the refined parameters, however, depends heavily on the parameter in question. In Fig. 5 ▸(*d*), normalized standard deviations (see caption for calculation of these) of the different refined parameters are shown. The size and atomic displacement parameters (ADPs, here shown as *B*_iso_) are greatly affected by noise and temporal differences, whereas the unit-cell dimensions are mostly unaffected. While the size parameter is a proxy for the ‘apparent’ correlation length in the PDF, *i.e.* the ‘extent’ of peaks in the PDF and thermal motion and thus the width of the peaks, the unit-cell dimensions are determined by the positions of the peaks. Considering ten consecutive 250 Hz exposures, the peak positions are unchanged, but as the noise level changes from frame to frame, the relative peak intensity can vary slightly (see Fig. S4), and this can affect the value of the refined size and ADP.

Now, another question arises concerning what the most suitable acquisition rate is, and this of course depends on the system in question. Reliable PDFs can be achieved with an acquisition rate of 250 Hz, and if the temporal changes of the sample are in the 250 Hz regime, this is clearly feasible, though at the cost of noise in the PDF, leading to greater uncertainty in particular refinement parameters. Here, it should be noted that DanMAX has the possibility of using a multi-layer monochromator (MLM) in place of the double-crystal monochromator (DCM), which was used for the data presented here. The MLM accepts a wider energy bandwidth and increases the flux at the sample position approximately fiftyfold. With the monochromaticity of the beam being less important for total scattering on very small nanoparticles that show broad reflections, this should in principle result in 250 Hz MLM PDFs and 5 Hz DCM PDFs of equal quality by extrapolation.

Ideally, the acquisition rate should match the timescale of changes within the sample. In the present case on the formation of HfO_2_ nanoparticles, the number of data points during the period of most rapid change (0–5 s) seems sufficient at 25 Hz to resolve the changes occurring during this period (Fig. S3). However, if the timescale is unknown or one wants to be sure that no sudden changes and transitionary states are missed, collecting the data at 250 Hz and afterwards virtually summing to the desired exposure seems a suitable possibility (although the duty cycle of the detector will decrease to 75% at 250 Hz compared to 99.9% at 1 Hz), while ignoring the challenges imposed by the large volume of data for a long experiment.

## 4. Conclusions

X-ray scattering is a powerful tool to investigate the nucleation and growth of nanoparticles, in real time and with sub-second temporal resolution. Even in the case with non-crystalline starting materials, total scattering and pair distribution function analysis can reveal key parameters that can be exploited to gain phase, morphological and size control of nanoparticles.

We have built and installed a solvothermal batch reactor on DanMAX at MAX IV, Lund, Sweden, and on P21.1 at PETRA III, DESY, Hamburg, Germany. The reactor systems are available to general users on the beamlines for this type of study. The reactor consists of a pressurized fused silica tube with an inner diameter of 0.7 mm that sustains up to 250 bar of pressure and 723 K in temperature, thus encompassing most solvo- and hydro­thermal reactions for *in situ* PXRD and PDF studies.

## 5. Related literature

For further literature related to the supporting information, see Ashiotis *et al.* (2015 ▸), Coelho (2018 ▸), Juhás *et al.* (2013 ▸) and Kieffer *et al.* (2020 ▸).

## Supplementary Material

Additional data and analysis. DOI: 10.1107/S1600576723002339/gj5297sup1.pdf
