# Supplementary material for: A reactor for time-resolved X-ray studies of nucleation and growth during solvothermal synthesis
Source: J Appl Crystallogr. 2023 Apr 13;56(Pt 3):581–8. doi: 10.1107/S1600576723002339 (PMC10241040; doi:10.1107/S1600576723002339)
Supplement: Supplementary file 1 [file j-56-00581-sup1.pdf]

JOURNAL OF APPLIED CRYSTALLOGRAPHY

Volume 56 (2023)

Supporting information for article:

A reactor for time-resolved X-ray studies of nucleation and growth during solvothermal synthesis

Martin Roelsgaard, Magnus Kløve, Rasmus Christensen, Andreas Bertelsen, Nils Broge, Innokenty Kantor, Daniel Sørensen, Ann-Chrstin Dippel, Soham Banerjee, Martin Zimmermann, Philipp Glaevecke, Olof Gutowski, Mads Jørgensen and Bo Brummerstedt Iversen

Total scattering data measured at DanMAX were collected with a PILATUS3 X CdTe 2M detector with a photon energy of 35 keV. Total scattering data measured at P21.1 were collected with a PerkinElmer XRD1621 detector with a photon energy of 103 keV. The detector distance was calibrated with a NIST 660b LaB_6 standard using *pyFAI* (Kieffer *et al.*, 2020; Ashiotis *et al.*, 2015). The 2D TS data were azimuthally integrated with *pyFAI* and PDFs were converted using *PDFGetX3* (Juhás *et al.*, 2013). All refinements were based on the study reported by Christensen *et al.* (2021). Both real space PDF and PXRD refinements were performed in TOPAS v. 7 (Coelho, 2018). In PXRD, the background was modelled using a combination of a scaled background measurement and a 4th degree Chebyshev polynomial. The parameters were the unit cell parameters (with a fixed β angle), ADPs for Hf only, crystallite size (using the built-in macro called LVol_FWHM_CS_G_L, however, only with Lorentzian contribution). For real space PDF refinements the refined parameters were the unit cell parameters (with fixed β angle), ADPs for Hf only, crystallite size (using spherical dampening). Instrumental broadening/dampening were accounted for using the LaB_6 standard for PXRD and PDF, respectively.

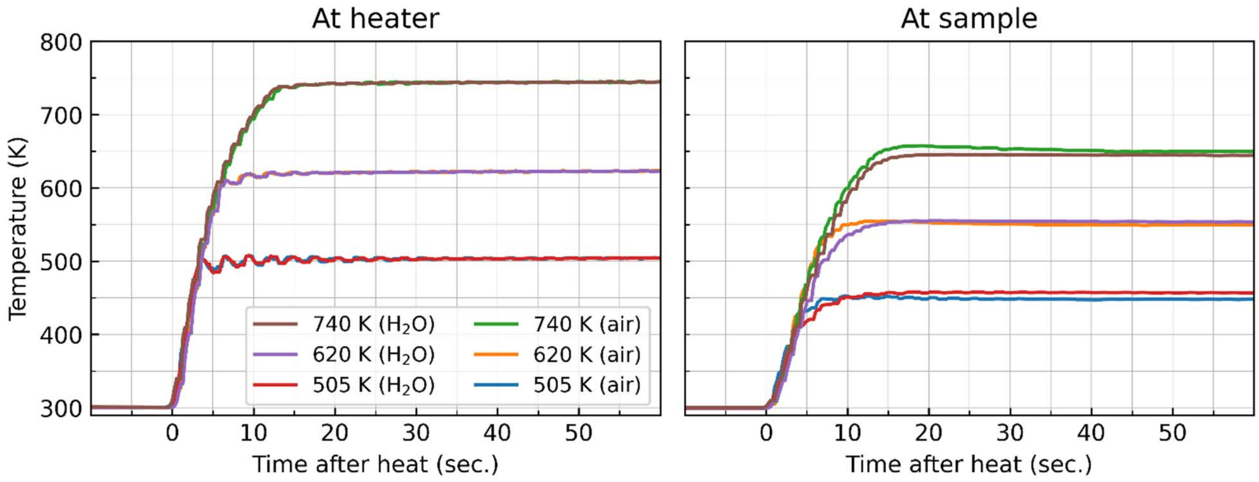

Figure S1. Heater calibration with a thermocouple situated in air (no external pressure) and in 250 bar pressurized water, inside of an Ø0.7 mm inner diameter sapphire tube. The legend denotes the target setpoint.

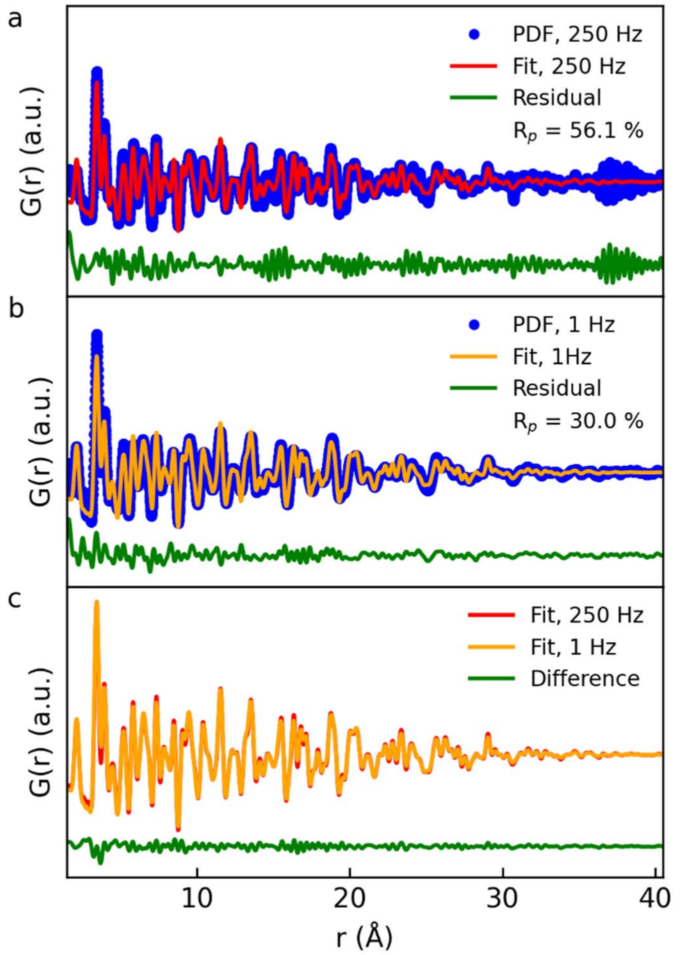

Figure S2. Structural refinements on a) 250 Hz PDF (R_{wp} = 56.1 %) and b) 1 Hz PDF (R_p = 30.0 %) after ~5.3 s. In c) the calculated PDFs from the fits in a) and b) are shown and the difference between them is calculated. Notably, the two calculated models are almost identical, and their difference curves are essentially featureless.

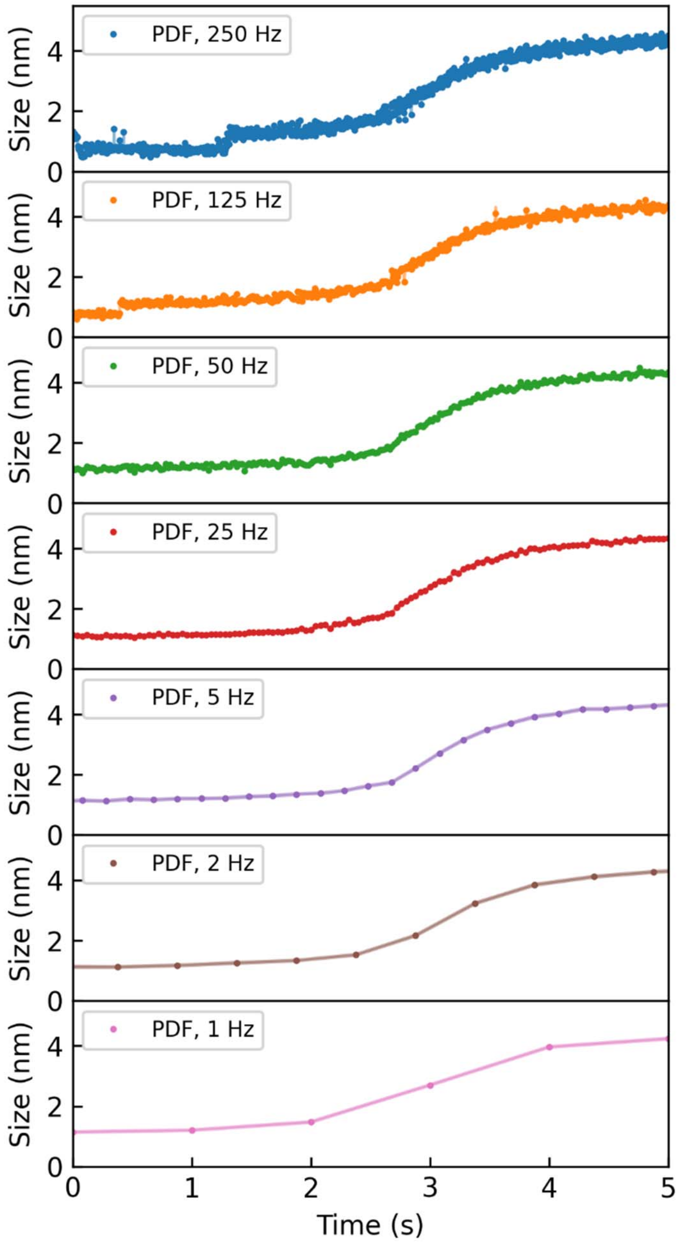

Figure S3. Refined average coherently scattering domain sizes from PDF refinements on HfO_2 formation with temporal resolutions from 250 Hz down to 1 Hz. Data are acquired at 250 Hz and virtually downsized by summing consecutive 250 Hz frames.

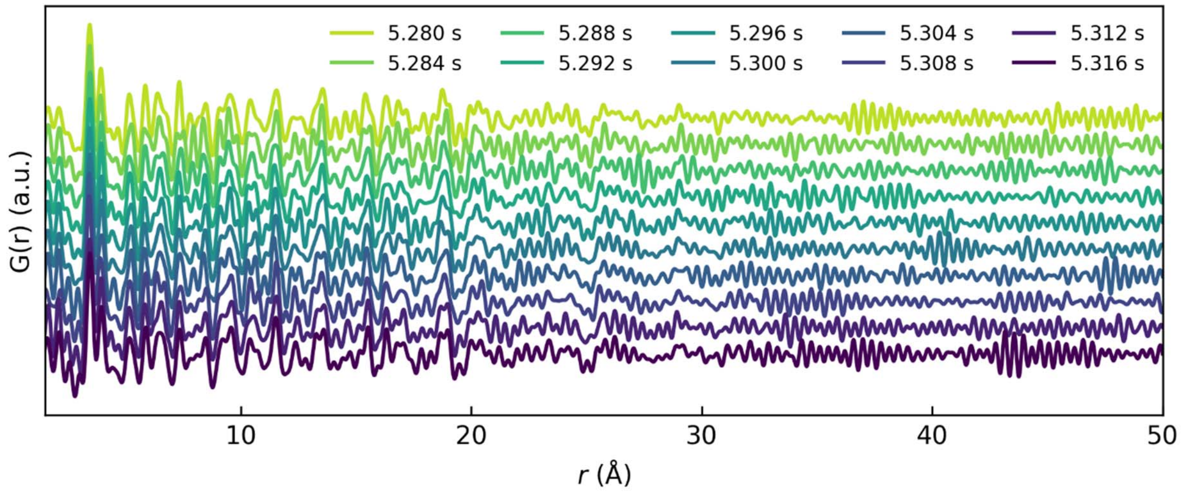

Figure S4. Ten consecutive 250 Hz PDFs showing changes in noise signal between consecutive exposures.

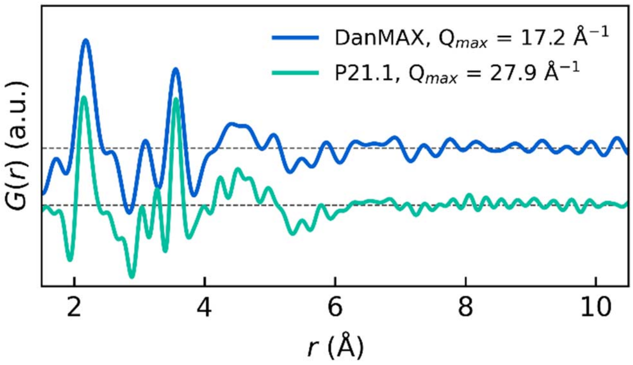

Figure S5. PDFs of $ZrCl_4$ precursors dissolved in water collected at DanMAX and P21.1.

SI references

Ashiotis, G., Deschildre, A., Nawaz, Z., Wright, J. P., Karkoulis, D., Picca, F. E. & Kieffer, J. (2015). *J. Appl. Crystallogr.* **48**, 510–519.

Christensen, R. S., Kløve, M., Roelsgaard, M., Sommer, S. & Iversen, B. B. (2021). *Nanoscale* **13**, 12711–12719.

Coelho, A. A. (2018). *J. Appl. Crystallogr.* **51**, 210–218.

Juhás, P., Davis, T., Farrow, C. L. & Billinge, S. J. L. L. (2013). *J. Appl. Crystallogr.* **46**, 560–566.

Kieffer, J., Valls, V., Blanc, N. & Hennig, C. (2020). *J. Synchrotron Radiat.* **27**, 558–566.
